# Molecular Mechanisms of Potato Plant–Virus–Vector Interactions

**DOI:** 10.3390/plants14152282

**Published:** 2025-07-24

**Authors:** Roza Kenzhebekova, Alexandr Pozharskiy, Kamila Adilbayeva, Dilyara Gritsenko

**Affiliations:** 1 Laboratory of Molecular Biology, Institute of Plant Biology and Biotechnology, Almaty 050040, Kazakhstan; rozakenzhebekova344@gmail.com (R.K.); aspozharsky@gmail.com (A.P.); kamila_1811@mail.ru (K.A.); 2 Department of Molecular Biology and Genetics, Al Farabi Kazakh National University, Almaty 050040, Kazakhstan; 3 Research Center AgriBioTech, Almaty 050040, Kazakhstan

**Keywords:** potato, PVY, PLRV, PVX, vector, transmission, plant response

## Abstract

Viral infections and their vector dynamics pose a major threat to potatoes (*Solanum tuberosum* L.) worldwide, urgently needing an integrated understanding of the molecular and ecological interactions in this tripartite system. This review describes the major potato viruses, namely potato virus Y (PVY), the potato leafroll virus (PLRV), and potato virus X (PVX), with an emphasis on their infection and replication strategies in plants, as well as their movement within them. It also discusses plant responses to these viruses by uncovering RNA silencing, resistance (R) genes, and hormonal signaling. The complex dynamics of virus–vector interactions are discussed, considering the modes of transmission-persistent, non-persistent and semi-persistent—the role of viral proteins such as HC-Pro in determining vector specificity and adaptations in vectors that facilitate virus dissemination. This article discusses how vectors select potato plants, with an emphasis on the role played by plant-excreted volatiles and vector-applied saliva in plant defense. It also discusses host genes that contribute to vector resistance. This review provides an overview of the interactions between potato plants, viruses, and vectors and shows how viruses influence plant–vector interactions, the molecular pathways shared, and the altered gene expression profiles due to these interactions. The review offers an integrated perspective essential for developing sustainable and precise control strategies against potato viral pathogens under changing climatic conditions.

## 1. Introduction

The potato (*Solanum tuberosum* L.) is the most important non-grain food crop in the world, and it is a key component of food and economic security. Its domestication is thought to have begun in the rugged highlands of the Andes Mountains of South America around 8000 years ago, and since then, it has been widely bred worldwide [1,2,3,4].

According to the Food and Agriculture Organization (FAO)’s data, in 2023, potatoes were cultivated on an area of 16,799,108 hectares worldwide, and global production reached 383,082,607.38 tons. The largest producing countries are China, India, Russia, Ukraine, and the United States [5]. Statistics show that despite the reduction in harvested area, potato production, in general, around the world tends to increase, which indicates an increase in yield. This may be due to the use of more productive varieties, improved agricultural technology, and optimization of cultivation [5].

One of the important aspects of increasing farming efficiency is the study and development of new technologies for crop protection against infectious diseases. Along with fungal infections, viruses pose a significant threat for potato growth and production. Potato plants are often attacked by various infectious viruses, among which the most economically significant are the potato leafroll virus (PLRV, family: *Solemoviridae* and genus: *Polerovirus*), potato virus A (PVA, family: *Potyviridae* and genus: *Potyvirus*), potato virus S (PVS, family: *Betaflexiviridae* and genus: *Carlavirus*), potato virus X (PVX, family: *Alphaflexiviridae* and genus: *Potexvirus*), potato virus Y (PVY, family: *Potyviridae* and genus: *Potyvirus*), and potato virus M (PVM, family: *Betaflexiviridae* and genus: *Carlavirus*). In particular, PLRV, PVX, and PVY have become a global threat to healthy seed potato production systems among the more than 50 viruses affecting potato crops worldwide [6,7,8,9,10,11,12,13,14,15]. Potato diseases caused by viruses pose a serious threat to potato production worldwide, causing significant economic losses and affecting food security. Climatic factors, including temperature, precipitation, and humidity, influence the status of insect vectors, thereby facilitating the spread of viruses in potato crops, especially under climate change [16,17]. Therefore, thorough control of viral infections is of great importance to maintain yield and production stability [18].

The adequate response to the threat of viral infections requires a comprehensive understanding of their biology and mechanisms of the interactions with the host plant. The studies of virus–vector–host relationships at the molecular level suggest new ways to control the spread of viral infections of plants, including potatoes. The role of the host in these relationships is determined not only by the presence or absence of the corresponding genetic factors of susceptibility and/or resistance but may also depend on the age and physiological state of the host plant. In many cases, plant resistance can be determined by sufficient host maturity (mature plant resistance) [19]. For a successful infection, the plant must be at a developmental stage in which its tissues are not only sufficiently susceptible to viral particle penetration but also permissive to the free movement of the virus, ensuring its availability for uptake by aphids [20]. In addition, certain molecules of the host plant itself also play a role in the infection process [19].

Since 80% of plant viruses are spread by insect vectors, the role of vectors in these relationships is of particular interest [21]. For example, knowledge of the transmission characteristics of a virus allows one to estimate the length of the latency period required for the virus to become capable of infecting other plants via the insect body.

The ability of a vector to transmit a virus is determined by genetic factors and specific molecular interactions between the virus and the vector itself. Strain-specific genetic changes can help viruses evade plant immune responses, altering their ability to infect host plants [22,23,24,25,26].

Changing climatic conditions also facilitate the successful dissemination of viral vectors and their establishment in regions that were previously unfavorable for them [27,28,29]. For example, an increase in temperature can accelerate the incubation period and reduce latent periods of the virus in the vector′s body. At higher temperatures, the motility and reproductive activity of viruses increase; this is because the viruses reach the salivary glands of the vector faster, which increases the frequency of plant infection. High temperatures also accelerate the manifestation of systemic infection in the plant organism [30,31,32].

An increase in air humidity facilitates the viability, reproduction, and mobility of many insect vectors and generally changes their behavior. An increase in precipitation facilitates the spread of weeds, which can serve as reservoirs of viruses. Rainfall facilitates the deposition of vectors from air currents onto fields, and more extreme weather conditions can damage plant tissue, providing a direct route for infection. Fluctuations in humidity (alternating wet and dry periods) increase the risk of infection with other (non-viral) diseases, which contribute to the weakening of the plant’s immune defenses [29,30].

Changes in the length of daylight can affect the activity of vectors and the life cycles of viruses. Wind facilitates the passive transport of flying vectors over considerable distances, including new territories [33,34].

Soil texture and quality can affect plant health and, as a result, susceptibility to viral infections. High levels of fine soil particles, such as clay and silt, can affect the microclimate in the soil and promote prolonged survival of the vectors carrying the viruses. In addition, high levels of available nitrogen and phosphorus in such soils stimulate the growth of tender plant tissues (young shoots and leaves), which in turn attract even more vectors [35]. Planting density is also one of the manageable factors that can significantly reduce or increase virus infestation through its effect on aphid dynamics. Wider spacing between plants increases the contrast between vegetation and bare soil, which attracts more winged insect vectors [36]. Overhead irrigation of potato plants increases humidity on foliage and in the crop canopy, creating favorable conditions for aphid activity and, consequently, increasing the risk of viral transmission [37].

In addition to physical environmental factors, there are also biological factors, including the presence of co-infections, commensal microorganisms, and adjacent plants [38,39]. All of these factors can lead to changes in disease manifestation. Thus, a better understanding of the interactions at all stages of the process, as well as the precise mechanisms of viral transmission, is important for development of more effective strategies to combat viral diseases in the future.

This review provides a comprehensive analysis of the molecular mechanisms of plant interactions with viruses and their vectors. Using PVY, PLRV, and PVX as examples, the most common potato viruses [7,40], the diversity of transmission strategies, host plant defense mechanisms, dynamics of virus–vector interactions, and novel molecular approaches to control these interactions are discussed. The review also highlights key pathways and targets for future research and integrated management of virus diseases.

## 2. Characteristics of the Most Common Potato Viruses

The spread of viruses in plants occurs due to complex interactions between the virus itself and the host plant, as well as between the vector and the plant, which ultimately leads to successful infection. The situation becomes even more complicated in the case of vegetative propagation, which is widely used in potato cultivation [41,42]. This method of reproduction contributes to the accumulation of viruses in plant tissues over several generations, which seriously complicates the fight against diseases. In addition, secondary viral infections can worsen the situation. When a plant is simultaneously infected with several viruses, a synergistic effect often occurs, in which the symptoms of the disease are much more severe than with infection by a single virus [38].

Among the viruses that affect potatoes, the most common and difficult to control are PVY, PLRV, and PVX [6,7,8,9,10,11,12,13,14,15]. The general comparison of three potato viruses is shown in Table 1.

### 2.1. Potato Virus Y (PVY)

PVY, belonging to the *Potyviridae* family and the *Potyvirus* genus [43], is one of the most economically significant and widespread viruses affecting potato. This virus is transmitted in various ways, spreads rapidly in agrocenoses, and causes significant yield reduction—up to 85% yield losses [38]. Transmission pathways include dissemination by the insect vectors, primarily aphids such as *Myzus persicae* (peach green aphid) and *Macrosiphum euphorbiae* (order: Hemiptera and family: Aphididae) [69,73]. The efficiency of PVY transmission via aphids may vary depending on the aphid species—more than 50 aphid species are capable of transmitting the virus [70,74]. Vegetative and mechanical paths of transmission of the virus are also known [70,71]. PVY can persist in alternative host plants, such as weeds and other members of the *Solanaceae* family, such as tomatoes, peppers, and eggplants [75]. This creates a natural reservoir of infection and significantly complicates virus control. In potatoes, the virus causes symptoms such as tuber necrotic rings (PTNRD), mosaicism, chlorosis, leaf deformation, and yield reduction. Depending on the PVY strain, the severity of symptoms can vary significantly [70,71]. PVY virions have a filamentous and flexible structure with helical symmetry, measuring 730–740 nm in length and 12 nm in diameter [53]. The viral genome consists of single-stranded positive-sense RNA (+ssRNA) that is covalently linked at the 5′ end to a viral protein (VPg) and contains a poly(A) tail at the 3′ end of the chain. The size is 9.7 kb [53,54]. The PVY genome contains a single large open reading frame (ORF) and an additional short out-of-frame (+2 translational frameshift) ORF [14]. The main ORF encodes a large polyprotein, which is subsequently cleaved by viral proteases into several functional proteins: P1, HC-Pro, P3, 6K1, CI, 6K2, VPg, NIaPro, NIb, and CP. Among them are three proteases, P1, HC-Pro, and NIa-Pro. The P1 protein is a serine protease responsible for the autocatalytic cleavage of the polyprotein [76]. In addition, it plays a role in the suppression of RNA interference and helps the virus adapt to different host plant species [53]. HC-Pro (helper-component protease) is one of the most well-studied and multifunctional viral proteins. It plays a key role in virus transmission by aphids, facilitating its movement within the insect body and enabling non-specific plant-to-plant transmission [44,76]. HC-Pro binds the capsid protein of the virus to receptors in the aphid mouth apparatus, according to the mechanism of the ‘bridge hypothesis’, thus ensuring efficient virus transmission. In addition, HC-Pro actively suppresses plant immune defense by inhibiting the RNA interference mechanism. It binds to small interfering RNAs (siRNAs), preventing their incorporation into the RISC (RNA-induced silencing complex) and suppressing the formation of secondary siRNAs. This disrupts post-transcriptional gene silencing (PTGS) and attenuates the antiviral response of the plant. The protease activity of HC-Pro is also required for the autocatalytic cleavage of the PVY polyprotein into functional domains. In addition, HC-Pro interacts with other viral proteins such as the capsid protein (CP) and the cylindrical inclusion (CI) protein, which promotes intracellular and systemic spread of the virus throughout the plant [44].

The P3 protein is involved in the formation of viral replication complexes (VRCs), specialized membrane structures that ensure efficient replication of viral RNA. It acts as an anchor for the replication complex due to interactions with other viral proteins, such as 6K2 and NIb (nuclear inclusion b), as well as the host plant proteins, determining the level of viral replication and motility [64]. The 6K1 protein is a small protein (~6 kDa) embedded in the endoplasmic reticulum (ER) membrane, where it contributes to membrane curvature and structural reorganization. Although 6K1 has no enzymatic activity, it plays a structural and supporting role in the early stages of the formation of the VRC. Conversely, the protein 6K2 is more directly involved in the formation of vesicular structures necessary for viral replication [77,78].

The CI protein has helicase activity and is involved in RNA replication and intercellular movement of the virus through plasmodesmata [79]. It forms characteristic spindle or helical inclusions near the plasmodesmata, and its ATPase activity is required for the unfolding of viral RNA secondary structures during replication and for active movement through the symplast [65,79].

VPg (viral protein genome-linked) is involved in the initiation of replication and interacts with cellular factors, particularly the cap-binding factor eIF4E (eukaryotic translation initiation factor 4E), to initiate translation of viral proteins and facilitate systemic movement of the virus in plants [56,57].

NIa-Pro (nuclear inclusion a-protease) is a cysteine protease that cleaves the polyprotein and regulates viral replication [80,81]. NIa-Pro not only mediates proteolytic cleavage but also interacts with VPg and possibly with plant ubiquitin-related pathways to provide temporal control of protein expression during infection [81]. NIb is an RNA-dependent RNA polymerase that catalyzes the synthesis of viral RNA [82,83,84].

The CP forms the capsid of the viral particle, provides protection for the RNA, and is involved in intercellular movement and transmission of the virus [79]. CP interacts with HC-Pro and CI to facilitate long-distance systemic movement and transmission of the virus by aphids. In addition, CP can suppress RNA interference in plants in conjunction with HC-Pro [44].

The additional ORF, known as the Pretty Interesting Potyviridae ORF, is translated into a shorter protein, P3N-PIPO or simply PIPO [62,66,77,79]. PIPO is expressed via a polymerase slippage mechanism, which results in a frameshift. In this process, the viral RNA-dependent RNA polymerase slips on a conserved GAAAAAA (GA_6_) motif or a similar sequence within the P3 protein coding region, resulting in the addition of one nucleotide and a −1 frameshift relative to the P3 frame [66,81]. The P3N-PIPO protein is localized in the plasmodesmata [66,79]. This protein interacts with plant proteins such as PCaP1 (a plasma membrane-associated protein that binds cations), as well as with other factors associated with the plasmodesmata, thereby facilitating the intercellular movement of PVY [66]. Viral RNA replication occurs in the cytoplasm of the host cell with the participation of the viral protein NIb, which recognizes and binds to the 3′ end of the viral genome [83]. Plant molecular chaperones, in particular NbHsc70-2, play an important role in this process, maintaining the virus in its active form and facilitating its spread in the plant [85]. The virus itself moves through the phloem of the plant in two directions: from infected leaves to tubers and from the infected mother tuber to young shoots [67,86] (see Table 1).

Like most RNA viruses, potato virus Y (PVY) has a high level of genetic diversity. The main strains are PVY^O^, PVY^n^, and PVY^c^, and at least nine recombinant variants have been identified to date, resulting from genetic exchanges between the PVY^N^ and PVY^O^ strains [30,87,88,89]. In Europe and North America, the most widespread of these new strains are PVY^NTN^ and PVY^N-Wi^ [90]. Differences between these strains are associated with the number of recombination points in each genome [86,91], serological reactivity [92], and the features of systemic movement of the virus in the host plant [93]. Recombinant isolates of the PVY^NTN^ strain contain three recombination points located in the genome regions encoding HC-Pro/P3, VPg, and CP [91]. Some isolates of this strain may contain a fourth recombination point in the region encoding the P1 protein [87,94]. PVY^N-Wi^ isolates include two groups depending on their recombination patterns: the first group has two recombination points (in the P1 and HC-Pro/P3 regions), and the second group, called PVY^N:O^ [70], contains only one recombination point in the HC-Pro/P3 region [86,91]. In addition, the new PVY^NTN^ and PVY^N^ Wi strains are distinguished by their virulence: both recombinants cause severe damage to tubers in susceptible varieties. Strain PVY^N-Wi^ causes tuber cracking [86], while PVY^NTN^ causes potato tuber necrotic ringspot disease (PTNRD) both reducing yield and product quality. General symptoms of PVY in potato include leaf mosaic, vein necrosis, leaf crinkling, and stunting [87].

### 2.2. Potato Leaf Roll Virus (PLRV)

Another common virus, PLRV, belongs to the *Polerovirus* genus of the Solemoviridae (formerly *Luteoviridae*) family [45,46]. It is transmitted by various aphids that feed on potatoes, primarily the peach aphid (*Myzus persicae*) [46,47]. PLRV is a phloem-restricted virus, meaning that its infection cycle occurs exclusively in the cells of the plant’s vascular system [48]. The virus particles are icosahedral in shape and about 24 nanometers in diameter. The capsid is formed by CP, which protects the viral genome and is involved in recognizing plant phloem cells [46,48]. In addition, CP is involved in the initial stage of virus interaction with the aphid’s oral structures, especially at the stage of non-specific attachment of the virus to the insect [46,49]. The PLRV genome is represented by a single-stranded positive-sense RNA (+ssRNA) of 5.3–5.7 kb in length [45,46,58,95]. At its 5′ end is VPg. It is thought to be involved in the initiation of viral RNA replication, as is usually characteristic of other members of the *Solemoviridae* family, although its precise function within PLRV has not yet been fully explored [57,96]. Another difference is that the PLRV genome lacks a 5′ cap and a 3′ poly(A) tail, suggesting the involvement of VPg in translation initiation and RNA stabilization [57,58]. However, direct interaction of PLRV VPg with host translation initiation factors such as eIF4E has not yet been experimentally confirmed [97]. On the surface of the viral capsid, there is also a protein (RTP; read-through protein) resulted from the read-through translation ignoring the stop codon [58]. Its C-terminal region is responsible for efficient movement of the virus within the plant, tissue specificity, and development of disease symptoms [48]. The N-terminal part of the RTP is necessary for its incorporation into the virion and plays a key role in virus transmission by aphids, as well as in interaction with proteins of aphid symbiotic microorganisms [58].

Among the proteins that are not part of the viral particle, P0 is especially important—it actively suppresses one of the key defense mechanisms of the plant, RNA interference [63,98]. In addition, P0 blocks signaling pathways associated with jasmonic acid (JA)—a hormone that is usually activated when attacked by pests or diseases. All this helps the virus “trick” the plant’s immune system. Another viral protein, P1, plays a similar role, participating in the copying of viral RNA. It is also able to suppress JA-mediated signaling pathways, thereby facilitating the spread of the virus [72]. Interestingly, P1 can turn into another protein—P1-P2—if a frame shift occurs during genome reading [96]. This fusion protein works as an enzyme (RNA-dependent RNA polymerase) and is necessary for the creation of new copies of the virus [96,99]. Another significant protein is P3a. It helps the virus move long distances through the plant’s vascular system. Although its mechanism of action is not fully understood, mutations in the P3a gene impair systemic infection, indicating its critical function in virus dissemination [58,68,100]. Recent studies suggest that the P7 protein (ORF7) may also be involved in the process of suppressing plant defenses. It may affect signaling pathways associated with another hormone, ethylene (ET), which is also activated in response to stress. If this pathway is blocked, the plant becomes more vulnerable and is more easily infected [45,58,72].

The entire process of PLRV reproduction occurs exclusively in the phloem tissues, the part of the vascular system responsible for the transport of nutrients [45]. Viral replication is strictly controlled, and even small changes in its genome can affect the ability to reproduce and transmit to other plants [45,58]. PLRV is transmitted through infected tubers or via aphids [69,72]. The severity of the symptoms depends on the potato variety and the presence of other viruses. The disease is generally accompanied by leaf curling, slow shoot growth, and chlorosis. All these symptoms seriously reduce yields and impair the quality of tubers [45,58,101].

### 2.3. Potato Virus X (PVX)

Another widespread virus that affects potatoes is PVX, which belongs to the genus *Potexvirus* of the *Alphaflexiviridae* family. The viral particles are filamentous and flexible, thread-like with helical symmetry, 515 nm long, and 13 nm in diameter [50,51,52]. Transmission does not require the participation of vectors and occurs through mechanical damage to tissues—by tools, hands, clothing, contact with other plants, or exposure to physical factors. The virus’s genome consists of a single-stranded molecule of positive polarity (+ssRNA) with the length of approximately 6.4 Kb. The genome contains five ORFs encoding the proteins RdRp, TGBp1, TGBp2, TGBp3, and CP [50,51,52]. The RdRp protein is responsible for synthesizing new viral RNA strands using the viral genome as a template. It also interacts with other viral proteins and host plant components to form a replication complex, which is essential for efficient viral replication [52]. TGBp1 (triple gene block protein 1, also known as P25) is the first of three proteins that make up the so-called “triple gene block” (TGB), which plays a key role in viral movement between plant cells. TGBp1 can interact with host proteins, plasmodesmata, and cytoskeletal elements. In addition, it has the ability to suppress RNA interference [55,102]. TGBp2 (triple gene block protein 2) is a membrane-associated protein that helps transport the viral RNA complex into plasmodesmata. It is embedded in internal cell membranes, particularly the endoplasmic reticulum. TGBp2 interacts with TGBp3 (triple gene block protein 3) and promotes the formation of transport vesicles that direct the virus to intercellular junctions [60]. TGBp3 delivers the viral complex directly to plasmodesmata and is involved in organizing membrane structures necessary for virus movement within the plant. It also plays an important role in coordinating intracellular transport of viral components [102,103].

The CP encapsulates viral RNA, forming stable virions. In addition to virion assembly and protection of RNA from degradation, the CP also promotes virus movement within the plant [61]. In PVX, the CP interacts with TGB proteins and plant cytoskeletal components and allows the virus to travel long distances through the phloem and establish systemic infection [61,102,104].

The process of virus replication can occur in different cells and tissues of the plant. The virus spreads systemically throughout the plant and can be retained during vegetative propagation. Infected plants are characterized by chlorosis, mosaicism, a reduced leaf size, and sometimes apical necrosis and necrotic lesions of tubers. PVX also synergizes with other viruses, resulting in increased severity of symptoms, leading to significant yield losses [52,61,100].

## 3. Viral Strategies for Successful Transmission via Vectors

**Figure 1:**
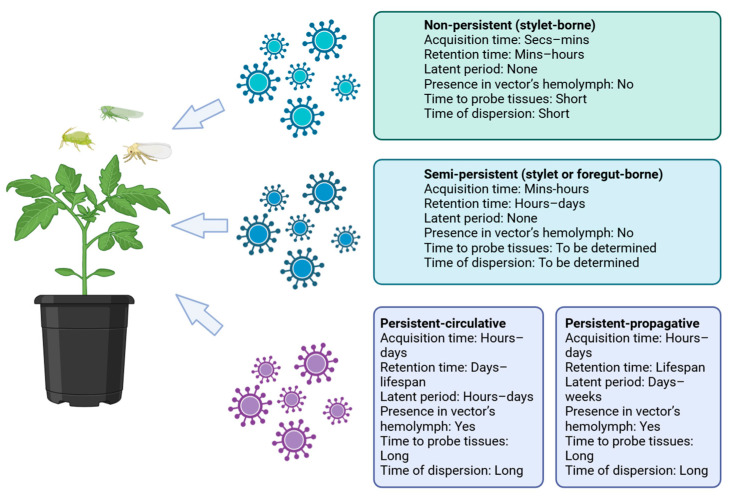
Transmission characteristics of insect vectors [105,109,110].

Plant viruses can use a variety of routes of transmission, but the most common is through insect vectors [69]. Virus–vector relationships are classified into non-persistent, semi-persistent, and persistent forms [105]. Examples of non-persistent transmission include potyviruses such as cucumber mosaic virus (CMV), which are transmitted by aphids [106]. Semi-persistent transmission involves closteroviruses such as beet yellows virus, which are also spread by aphids and whiteflies [107]. Persistent transmission includes geminiviruses (tomato yellow leaf curl virus), which are transmitted by whiteflies, and bunyaviruses (tomato spotted wilt virus), which are transmitted by thrips [108]. In each of these, the virus interacts differently with the vector and host plant. In non-persistent transmission, the virus persists in the insect’s mouthparts (mostly the stylet) for minutes to hours. Semi-persistent transmission is characterized by the virus being retained in the anterior digestive tract of the insect (in the foregut or salivary glands) for hours or days. Persistent transmission occurs when the virus passes through the insect’s intestines into the hemolymph and salivary glands, allowing it to be transmitted throughout the life of the vector. This type of transmission can be divided into circulative (without replication in the vector) and propagative (replicates within the vector). The most common potato viruses PVY and PLRV are characterized by non-persistent and persistent modes of transmission, respectively [105,109,110] (Figure 1). PVX is transmitted mechanically through plant-to-plant contact, such as via wounds or contaminated tools, and is not known to be insect-transmitted [52].

The manipulation of the behavior and physiology of insect vectors by viruses in ways that optimize transmission has been documented by numerous studies [111,112]. In the case of PVY, it has been established that when transmitted horizontally, the virus indirectly affects the reproduction of aphids: it changes the plant in such a way that it becomes a more favorable environment for the development of aphids, which can lead to an increase in the number of infected individuals in subsequent generations [72,111,112]. Additionally, PVY exerts varying effects on different aphid species [113]. In *Myzus persicae*, which is the main vector for potato plants, the chemical composition of plant sap is altered under the influence of the virus, which leads to a change in the duration of feeding and, as a consequence, increases the efficiency of PVY transmission to 80% under experimental conditions [111]. Transmission of the virus from an infected plant to the vector is mediated by specific protein interactions: The DAG motif on the viral capsid protein binds to the PTK motif of the HC-Pro protein. After this, the KITC motif at the N-terminus of HC-Pro interacts with receptors on the aphid’s mouthparts, which facilitates attachment of the virus to the insect [44,70]. Thus, the HC-Pro protein acts as a “bridge” between the virus and the aphid, helping the virus particle enter a new plant. However, this mode of PVY virus transmission creates narrow genetic bottlenecks, i.e., insect-transmitted virus populations are the least genetically diverse. Nevertheless, vector-mediated transmission facilitates rapid fixation of new mutations in individual viral lineages, which may affect virus adaptation to field conditions [42].

PLRV is circulatively transmitted by *Myzus persicae*. Transmission is initiated when an aphid punctures the virus-infected plant, allowing the virus to enter the stylet and reach the foregut. After entering the aphid, PLRV binds to the receptors localized on the apical membrane of midgut epithelial cells to trigger clathrin-mediated endocytosis. Then, the virus enters the intestinal cells and moves into the hemocoel, from where it is distributed to the salivary glands. Once in the salivary ducts, it is transmitted to a new plant during the aphid’s next feeding [58,112].

Transmission of PLRV is regulated by the protein C1QBP, which limits virus dissemination within the aphid. C1QBP acts as a transmission barrier, reducing the accumulation of the virus in aphid tissues and limiting its penetration into the salivary glands, thereby decreasing transmission efficiency by approximately 1.7-fold [114].

Additionally, PLRV is able to alter the expression of genes associated with odor perception and foraging behavior in *M. persicae*, which increases the likelihood that aphids will linger on infected plants, thereby facilitating the spread of the virus [115].

In addition, studies have shown that PLRV is capable of causing profound reorganization of vector gene expression.

PLRV infection induces the expression of 134 *M. persicae* genes, with upregulation of cytochrome P450 and cuticle genes reinforcing the vector’s defenses and downregulation of heat shock proteins (HSPs) and immune response genes compromising stress tolerance [114,116]. The virus also lowers titers of the *Buchnera aphidicola* symbiont, which likely compromises aphid metabolism and reproductive performance [116]. PLRV-infected aphids feed longer on infected plants, increasing the likelihood of virus transmission [117]. Unlike PVY, PLRV-infected plants emit higher levels of volatile organic compounds (limonene, pinene, cadinene, caryophyllene, and α-humulene), attracting *M. persicae* and facilitating virus dissemination. Infected aphids also have reduced motility, facilitating local spread of PLRV [72].

Phytophagous insects often use chemical cues to locate and select host plants [118]. This opens the possibility for viruses to manipulate vector behavior by influencing the processing of olfactory and/or gustatory cues [119]. In insects, these cues are processed by soluble binding proteins found in the olfactory and gustatory organs [120]. Chemosensory proteins (CSPs) and odorant binding proteins (OBPs) are conserved among insects and have been identified as the major soluble proteins in insect sensillary lymph [121]. Their conservation highlights the importance of host plant selection and is exploited by viruses to alter vector preference by altering CSP and OBP expression [122]. Existing studies involving manipulation of insect vectors and plant viruses have considered only one host plant species, limiting our understanding of the mechanisms of action [123]. Therefore, there is a need for extensive studies involving multiple plant species, since vector manipulation may be species-specific and most plant viruses have a broad host range [124].

Such changes in vector behavior and physiology may be the result of coevolution between viruses, vectors, and insects, where viruses are selected for the ability to manipulate vectors and vectors are selected for resistance to the negative effects of viral infection [115].

Climate conditions can also affect the vector-mediated spread of potato viruses. A warm climate promotes the growth of insect populations, leading to a wider virus distribution [16,17,41,69].

Unlike vector-transmitted viruses, PVX spreads exclusively through mechanical transmission, requiring direct contact between infected and healthy plants rather than relying on insect vectors [52].

## 4. Vector–Host Interactions in Plant Virus Transmission

Vector–host interactions, particularly in the context of plant viruses, are complex, multifaceted, and dynamic. Studies have demonstrated that plant viruses can influence vector behavior and physiology, increase per-vector transmission efficiency, and modify ecosystem processes. One basic concept that can be applied to the study of vector–host interactions is the vector manipulation hypothesis (VMH) [110,112,125].

According to this hypothesis, infected plants modulate insects’ feeding habits, and more specifically, viruses promote piercing and feeding on infected plants, which raises the probability of their infection and spread [72,110,112,125]. Existing studies of viruses affiliated with the *Luteoviridae* family, such as PLRV, and aphid vectors have demonstrated that virus infection affects the combination of developmental rate and reproductive potential of aphids [72,117].

Similar mechanisms of interaction have been identified in other plant–virus–vector systems. For example, the cucumber mosaic virus (CMV) is able to alter the behavior of aphids: it attracts insects to infected plants but at the same time shortens their feeding time. This strategy facilitates efficient transmission of the virus in a non-persistent form [125,126]. The tomato yellow leaf curl virus (TYLCV) behaves in a similar way, positively affecting the viability and feeding of its main vector, the whitefly *Bemisia tabaci*. This, in turn, facilitates more active spread of the virus among tomato plants [127,128]. The barley yellow dwarf virus (BYDV) on cereal crops accelerates development and increases the reproductive activity of the aphid *Rhopalosiphum padi*, which lead to rapid growth of the insect population and increased transmission of the virus [129].

One of the key mechanisms underlying these interactions is the change in the profile of volatile organic compounds (VOCs) emitted by the plant in response to virus infection [125,130]. Infected plants begin to produce a wide range of volatiles, including terpenes, sesquiterpenes, green leaf volatiles (GLVs), fatty acid derivatives, aromatic and nitrogen-containing compounds, and volatile phytohormones such as methyl salicylate and methyl jasmonate. These virus-induced volatiles can influence vector behavior in various ways, attracting, repelling, or having no detectable effect [72]. Most importantly, GLVs, sesquiterpenes, and terpenoids emitted by CMV-, BYDV-, and PLRV-infected plants attract aphids that do not carry the virus, thereby stimulating their initial feeding on infected plants over healthy ones [72,112]. For example, *Myzus persicae* and *Aphis glycines* were more likely to prefer CMV-infected pepper plants; this was attributed to the elevated ethylene levels in these plants. This change in the volatile background influences the insects’ behavioral preferences and facilitates virus transmission. Moreover, such interactions can alter not only the efficiency of virus transmission but also the ecological relationships between the host plant and its surrounding ecosystem [125,130].

These interactions can affect not only the populations of individual species but also broader processes in ecosystems. For instance, one (or multiple) virus can differentially impact vector fitness and behavior, complicating the underlying transmission landscape [130,131]. The coevolution of viruses and their vectors implies that these interactions are under natural selection, favoring characteristics that allow for increased efficiency in viral spread [111,113].

In addition to this theory, the vector–host relationship can be attributed to the fact that aphids can perform multiple brief feedings on plants that are not their primary hosts, which facilitates the spread of non-persistent viral infections for which entry onto the insect’s stylet is sufficient for dissemination [113,117,129].

Moreover, the presence of reservoir weeds (such as species of the genera *Brassica*, *Erodium*, *Sonchus*, *Plantago*, and *Raphanus*) also increases the ability of the virus to spread, even in the absence of potato plants. The risk of dissemination is also increased by the diversity of insect vectors that may not be direct vectors of potatoes but transmit the virus through random probes (*Diuraphis noxia*, *Phorodon cannabis*, and *Rhopalosiphum padi*) [58].

## 5. Potato Plant Responses to Virus Infections: Defense Mechanisms and Physiological Changes

### 5.1. General Defense Mechanisms

Pathogen–host interactions are complicated and include, on the one hand, the viral strategies of targeting host cell processes and, on the other hand, plant defense strategies against infections [132,133]. Plants have an extremely complex and flexible immune system that enables them to effectively resist viral infections [134,135,136]. Plant immune defense includes two levels. The first line of defense is based on the plant’s ability to recognize common molecular patterns characteristic of a wide range of pathogens, the so-called PAMPs (pathogen-associated molecular patterns) or MAMPs (microorganism-associated patterns) [134]. These molecules trigger a basic innate immune response, which can be considered as the plant’s “early warning system”. PAMPs (MAMPs) are highly conserved molecules that are characteristic of entire classes of pathogens and are recognized by the innate immune system of plants. PAMPs molecules induce a basal immune response, which adapted pathogens are able to evade by avoiding recognition by plant receptors [134,135]. The second line of plant immune defense consists of resistance proteins (R proteins) that recognize viral effectors directly or through associated host proteins. This mechanism of the immune response is usually strain- or species-specific and often results in a hypersensitive response (HR), a localized cell death limiting viral replication and movement [134,135,136,137]. Activation of R proteins initiates a cascade of intracellular signaling processes, one of the key components of which is the production of reactive oxygen species (ROS). These molecules have a dual nature: on the one hand, they can damage cellular structures, and, on the other hand, they act as signaling molecules that warn neighboring cells about the presence of a threat. Excessive accumulation of ROS is potentially dangerous, so antioxidant regulation mechanisms are activated in resistant plants [138,139].

For example, in resistant potato varieties, the antioxidant system is activated, maintaining the balance between the formation and neutralization of ROS. An increase in the activity of superoxide dismutase (SOD), catalase (CAT) and peroxidase (POD) enzymes was observed in the resistant plants, which effectively neutralize toxic compounds [140]. At the same time, a decrease in the level of malondialdehyde (MDA), a marker of damage to cell membranes, and an increase in the concentration of proline, a low-molecular-weight compound involved in the adaptation of cells to stress and in maintaining their structural stability, was observed [141].

### 5.2. Defense Against PVY

**Figure 2:**
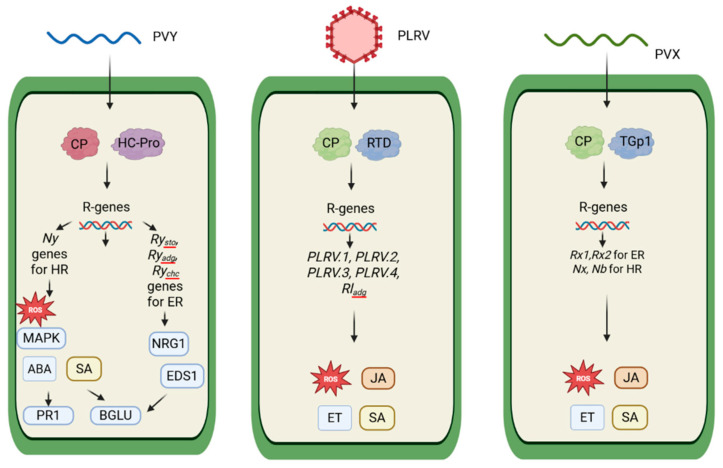
Simplified scheme of the R gene-mediated response to avr (avirulence factor) and defense mechanisms of potato against viruses [72,152,153,162,163,164].

To date, different defense mechanisms of potatoes against PVX, PVY, and PLRV have been studied (Table 2). Potato plants have two main types of genetic resistance to PVY: a hypersensitive reaction (HR) and extreme resistance (ER) [142]. The HR, controlled by the *Ny*-1 and *Ny*-2 genes, is characterized by the appearance of localized tissue necrosis at the site of virus entry, which prevents its systemic spread. However, *Ny*-1 loses efficacy at temperatures above 28 °C, allowing the virus to spread through the plant without visible symptoms. *Ny*-2 remains active at 28 °C, but its ability to limit infection depends on specific conditions such as the virus strain, the stage of plant development, and external factors such as temperature stress or co-infection [143]. The *Ry*_sto_, *Ry*_adg_, and *Ry*_chc_ genes provide ER by completely blocking virus replication without any visible symptoms. Unlike the temperature-sensitive HR, ER-type resistance works effectively even at high temperatures [144]. Interestingly, when two resistance genes, *Ry*_sto_ and *Ny*-1, are present in the plant at the same time, *Ry*_sto_ can override the HR triggered by *Ny*-1, so the typical necrotic response does not occur under these conditions (Figure 2) [145].

In addition to resistance genes (R genes), which limit viral infection, host susceptibility factors (S genes) play an equally important role in virus–host interactions. An example of such a protein encoded by S genes is eukaryotic translation initiation factor 4E (eIF4E) and its isoforms eIF4E1, eIF4E2, and eIF(iso)4E. This family of proteins recognizes the cap structure at the 5′ end of mRNA to initiate translation via a cap-dependent mechanism. Plant viruses, especially those of the *Potyviridae* family, use them as susceptibility determinants to achieve successful replication. Moreover, several viruses can simultaneously recruit two or more isoforms [165]; therefore, the knockout of a single eIF4E isoform is not sufficient to confer resistance [166,167]. At the same time, simultaneous knockout of several isoforms can lead to inhibition of plant growth or the emergence of hypersensitivity to other pathogens [168,169]. Studies have shown that the interaction between eIF4E and VPg is highly specific, as a single amino acid substitution in the susceptibility factor can completely disrupt binding [170,171]. Therefore, the CRISPR/Cas9-based targeted base editing method can be used to alter individual codons in the susceptibility factor to enhance resistance [172]. When PVY infects a potato plant, it activates a complex immune signaling network, including mitogen-activated protein kinase (MAPK) cascades, which play a crucial role in regulating defense responses. One of the key proteins involved in this signaling is StMKK6, a potato-specific MAPK. Upon infection, the expression of StMKK6 is significantly upregulated, and the protein accumulates in the nuclei of infected cells. This nuclear localization suggests that StMKK6 participates in the activation of transcription factors and defense-related genes necessary to mount an effective response against the virus [156].

Moreover, to the MAPK signaling cascade, salicylic acid (SA) plays a key role in potato resistance to PVY [155]. SA accumulation in infected tissues helps inhibit virus replication and enhances the HR, a localized form of programmed cell death that prevents the virus from spreading throughout the plant. SA signaling leads to PR1 (pathogenesis-related protein 1) and *BGLU* (*Glu-III*) expression, callose deposition, and cell wall reinforcement [162]. The relationship between SA and the StMKK6 signaling protein is confirmed by experiments: transgenic potato plants with impaired SA synthesis (e.g., due to expression of the NahG gene) exhibit reduced activity of the StMKK6 gene and, as a result, increased sensitivity to PVY infection and more severe disease symptoms [139,145,156,173]. While SA-mediated signaling and StMKK6 activation are central to HR induction, ER employs distinct immune components, including *NRG1* (*N requirement gene 1*) and *EDS1* (*Enhanced Disease Susceptibility 1*), to prevent virus replication and systemic spread without symptoms [162].

Additionally, an antagonistic relationship was found between the SA and gibberellin (GA) signaling pathways in potato plants. In the study, the tolerant potato variety Désirée demonstrated activation of the small RNA regulatory network, in particular miR167 and phasiRNA931, which suppress the expression of GA biosynthesis genes. This process was recorded as early as the third day after inoculation, before virus replication was detected. Thus, there is a suppression of growth-related processes for which gibberellins are responsible, and activation of plant defense reactions is initiated by SA [174].

A strong immune response to PVY includes activation of pathogenesis-related (PR) genes, especially PR-1b [142], a molecular marker of SA-mediated protection [155]. Increased PR-1b expression enhances both local defense responses and systemic acquired resistance (SAR), limiting virus dissemination and generally increasing plant immune readiness [173,174,175,176]. In addition, molecular studies have shown that during PVY infection, the expression of not only protein-coding mRNAs but also small RNAs (microRNAs, miRNAs), which play an important role in gene regulation, is altered [177]. This means that plant resistance is determined not only by specific “immune” genes but also by a broader complex of genetic and metabolic pathways. During infection, plants also produce virus-induced small interfering RNAs (vsiRNAs), which can suppress the expression of their own genes and thus modify the immune response. The studies revealed 88 functional interactions between miRNAs and mRNAs, confirming the important role of these molecules in plant defense against viruses [177,178].

The mechanism of RNA interference (RNAi) plays an important role in the formation of plant resistance to PVY. The RNAi process requires the formation of the RNA-induced silencing complex (RISC). Assembly of this complex involves DICER-like proteins (DCLs), Argonaute proteins (AGOs), and small RNAs such as siRNAs and miRNAs. The RISC then targets viral RNAs in the cell and cleaves them, thereby halting viral replication [179]. However, PVY contains a strong suppressor of RNA silencing, namely the HC-Pro protein. This protein binds to short interfering RNAs (siRNAs), preventing their incorporation into the RISC. The HC-Pro protein contains a central domain with a FRNK motif that directly binds to short interfering RNA (siRNA/miRNA) duplexes, keeping them inactive. The mechanism of action of HC-Pro involves competition with Dicer-like proteins (DCL2/DCL4) and Argonaute (AGO), which ultimately leads to disruption of RISC formation. By interfering with the RNAi pathway, HC-Pro suppresses DCL activity and reduces the expression of plant genes involved in gene silencing [44,179]. These strategies enable PVY to evade plant immune responses and spread systemically throughout host tissues [59,134,135,146].

In the study by Murtaza S. et al., it was shown that plant-mediated RNAi effectively suppresses MIF1 gene expression in *Myzus persicae* and increases aphid mortality. Transgenic potato lines expressing dsRNA against MIF1 provided up to 77% aphid mortality and a decrease in MIF1 mRNA levels by up to 21% [180]. Another study by Bahrami Kamangar, S. et al. showed that RNAi silencing of the aphid cuticular protein genes MPCP2 and MPCP1 significantly reduced their expression by 63% and 75%, respectively [181]. These examples confirm the potential of this strategy for protecting plants from aphids and the viruses they transmit.

### 5.3. Defense Against PLRV

Resistance genes to PLRV have been identified in potato plants, including Rladg, PLRV.1, PLRV.2, PLRV.3, and PLRV.4 [152] (Figure 2). One of the key genes is Rladg, which is associated with a resistant reaction against PLRV. This gene likely functions by recognizing specific viral components, such as the CP and RTD (read-through domain), and subsequently triggering host defense responses that limit viral replication and systemic movement through the phloem [153]. The presence of Rl_adg_ can be detected using the molecular marker RGASC850, which is widely used in marker-assisted selection for breeding PLRV-resistant potato varieties [13,154].

As with other viruses, the RNAi machinery in plant cells plays a key role in potato defense against PLRV. Studies have shown that silencing of essential viral genes such as CP and MP by RNAi effectively disrupts capsid formation and packaging of the viral genome, rendering the virus unable to spread systemically [147,148,182]. Infection with PLRV also activates SAR, which is partially mediated by siRNAs that bind viral RNAs and trigger their degradation prior to translation. Although RNAi mechanisms have been well studied in relation to several potato viruses, such as PVX and PVY, their application against PLRV is in its early stages. Despite successful examples of siRNA-based silencing of PLRV, additional research is needed to optimize RNAi strategies and integrate them into resistant potato breeding programs [147,148].

The most well-known viral protein of the *Polerovirus* genus involved in suppression of RNAi is the P0 protein [98,183]. Numerous studies have confirmed the involvement of P0 proteins of several economically important poleroviruses, including PLRV, in suppressing RNAi [98].

The P0 proteins of different poleroviruses share several common motifs and regions that are critical for RNAi suppression. These include the F-box-like motif, the G139/W140/G141 motif, and the conserved C-terminal region. Studies have shown that removal of the N-terminal portion of the protein is characterized by suppressor activity blockage, while removal of the C-terminal regions affects the level of systemic suppressor activity [183]. The mechanism of RNAi suppression by P0 proteins is their binding to the membrane-bound protein AGO1 and subsequent degradation of AGO1 via the autophagic pathway involving the endoplasmic reticulum [184,185,186].

PLRV has evolved additional mechanisms to suppress plant immunity, particularly by targeting JA and ET signaling. JA signaling plays a key role in resistance to necrotrophic pathogens and phytophagous insects [187], and its suppression by the virus weakens plant defenses against insect vectors, facilitating their successful colonization. Studies have also shown that PLRV infection increases aphid fecundity and settlement on the host plant [188]. Dysregulation of ethylene by viruses further enhances vector productivity [187].

PLRV proteins P0 and P1 block JA-dependent defense responses, while P7 inhibits ET signaling. These hormonal pathways play a key role in coordinating stress responses, regulating the antioxidant system, and maintaining ROS homeostasis. Their suppression weakens the plant’s resistance, disrupts the functioning of protective networks, and changes the ROS balance, creating favorable conditions for virus replication and its systemic spread [72].

In addition, studies have shown that PLRV, both alone and in combination with PVY, negatively affects the photosynthetic apparatus of potato plants. Infected plants exhibit a significant decrease in chlorophyll a and b content, with resistant varieties demonstrating smaller losses compared to susceptible ones. However, resistant varieties maintained higher carotenoid levels, which contribute to protection against photodamage and oxidative stress [189,190].

### 5.4. Defense Against PVX

PVX, like the other viruses mentioned, causes a complex chain of molecular and physiological reactions in plants. These interactions include RNA interference mechanisms, the HR, ER, and changes in the antioxidant system [51,149,150,151].

One of the ways in which the plant protects itself from PVX is RNAi. The DCL2, DCL4, AGO2, AGO3, and RDR6 genes play a key role in this process. Reduced expression of DCL2 and DCL4 leads to viral RNA accumulation and enhanced susceptibility to PVX infection [150].

Like other aggressive pathogens, PVX disrupts cellular homeostasis of ROS, which further weakens the plant’s defense mechanisms [151]. When PVX and PVY are combined, a synergistic effect develops, which increases the severity of symptoms and leads to more serious tissue damage [157,158].

Viral infections alter the hormonal homeostasis of plants. Abscisic acid (ABA) plays an important role in antiviral defense. In the case of PVX, ABA accumulation suppresses virus replication by activating the RNAi mechanism, whereas PVY infections are often accompanied by increased ABA levels in susceptible cultivars, highlighting the different pathogenicity mechanisms of these viruses [159].

In addition, a hormonal response in the form of a decrease in the level of active cytokinins was detected for viruses of the *Potexvirus* family. This decrease is associated with a change in the physiological state of the plant and the development of infection symptoms. The hormonal response of the NahG potato line to PVX infection is similar to that to PVY and is characterized by a decrease in SA levels. This results in a weakening of the HR mediated by the Nb resistance gene to PVX and, as a consequence, a failure to induce SAR [191].

Moreover, general shifts in hormonal balance, such as increased SA levels triggering SAR and modulated JA and ET levels, have also been recorded for PVX [187,191].

Endophytic bacteria also contribute to potato resistance to viral diseases. Induced systemic resistance (ISR) activated by endophytes is a key component of plant immune defense against PVX. Unlike systemic acquired resistance (SAR), ISR does not involve the accumulation of pathogenesis-related (PR) proteins. Instead, it primes the plant for enhanced defensive responses upon pathogen attack. Bacteria of the genus *Bacillus* stimulate the expression of defense genes, reducing virus replication. In addition, increased ribonuclease activity in endophyte-treated plants enhances viral RNA degradation, resulting in a reduced viral load. Experiments using qRT-PCR and ELISA confirmed that treatment with *B. subtilis* strains 26D and Ttl2 significantly reduced the virus concentration in potato leaves [160,161]. A similar effect on plants after *B. subtilis* application was observed for protection against PVY [161].

Both mechanisms, HR and ER, are triggered by *R* genes (Figure 2), with the HR limiting virus spread via localized cell death, and ER preventing virus replication [51]. Two important HR-modulating genes involved in this PVX reaction are *Nx* and *Nb*. One of the key factors triggering the HR is the P25 movement protein encoded by PVX. It acts as an inducer of local cell death, inhibiting the spread of the virus through tissues [142,143].

*Rx* genes play a major role in the process of formation of extreme resistance to PVX. Recognition of the viral capsid protein by the *Rx* receptor activates plant defense mechanisms. *Rx* genes were first identified in potato and described as key genes in the formation of durable resistance by recognizing the viral capsid protein and triggering a reaction that limits virus replication and prevents symptom development [163,164]. The major ER genes are *Rx*1 and *Rx*2. These genes encode NBS-LRR (nucleotide binding site–leucine-rich repeat) proteins, which recognize the PVX capsid protein and block replication early during infection. Unlike the HR, ER is effective against all tested PVX isolates without inducing necrosis [142,143].

*Rx* proteins function properly in the presence of SGT1 (a ubiquitin ligase) and HSP90 (heat shock chaperones). They interact with RanGAP, a nuclear regulator involved in resistance signaling. Mutations in *Rx* genes can extend their protective capacity, enabling them to defend against other viruses such as the poplar mosaic virus (PopMV) [192].

As with PVY, host S genes associated with the translation process, specifically eukaryotic elongation factor 1 (eEF1), have also been identified for PVX. The main function of this factor is the delivery of aminoacyl-tRNA (aa-tRNA) to the elongating ribosome in a GTP-dependent manner. In plant cells, the eEF1 complex consists of the eEF1A and eEF1B proteins [193]. The eEF1B protein, in turn, includes a structural protein (eEF1Bγ) and two nucleotide exchange subunits (eEF1Bα and eEF1Bβ). Studies have shown that eEF1Bα and eEF1Bβ interact with eEF1A, and eEF1A and eEF1Bβ interact with the PVX triple gene block protein 1 (TGBp1) protein. Thus, eEF1A and eEF1Bβ are thought to play critical roles in PVX replication and movement through physical interaction with TGBp1. Furthermore, studies of other RNA viruses have shown that eEF1A plays a key role in viral replication through interaction with viral RNA and/or viral RNA-dependent RNA polymerase (RdRp) [194,195,196].

## 6. Future Strategies and Research Priorities in the Context of Potato Virus Management Through Manipulation of the Virus–Vector–Host System

Studies of the molecular mechanisms of virus–vector–host relationships remain promising, as not all the details have been studied and are fully understood. Recent studies have demonstrated that potato viruses such as PVY and PLRV can modulate gene expression in their aphid vectors (*Myzus persicae*), affecting metabolic and immune-related pathways [197,198].

In the virus–vector interaction, promising areas of research are an in-depth study of gene expression in the vector organism, aimed at identifying factors that increase the efficiency of virus transmission; analysis of the mechanisms of development of vector resistance to the negative effects of viral infection; as well as the search for target proteins in the vector organism in order to limit viral transmission [198,199]. For example, studies have shown that the cuticular protein MPCP2 in *Myzus persicae* is critical for the transmission of PVY, and that silencing the expression of this protein reduces the efficiency of virus transmission by nearly 50%, suggesting its potential role as a target for virus control [181]. In addition, genetic studies of M. persicae populations have identified resistance mutations in sodium channel genes (*kdr* and *skdr*) that may influence both aphid responses to insecticides and viral infections [200].

In addition, hormonal regulation of potato resistance, modulation of plant signaling pathways by viruses, the possibility of using phytohormones to improve plant resistance, and the effect of mixed infections on hormonal status have not been fully studied [187,201,202]. Studies have shown the role of SA as an essential component of the hypersensitive response and its role in coordinating defense signaling pathways to limit virus spread; however, the interactions between SA and other phytohormone signaling networks remain incompletely understood and require further investigation [155,202].

Of particular interest are multiomic approaches (transcriptomics, proteomics, and metabolomics), which allow for the identification of key genes and metabolites of potatoes involved in the antiviral response [203,204]. Thus, metabolomic research methods allow us to identify characteristic changes in the composition of secondary metabolites associated with antiviral defense reactions of plants [204,205]. Another important discovery was that the Bacillus licheniformis strain POT1 activates polyphenol biosynthesis pathways and increases potato resistance to the alfalfa virus, highlighting the role of secondary metabolites in antiviral defense [206]. Recent transcriptomic and small RNA analysis of PVY-infected potato cultivars revealed significant changes in the expression of many genes as well as in the miRNA profile, shedding light on antiviral mechanisms and potential resistance markers [207]. Further studies of the role of small RNAs in the formation of SAR are also relevant. TAS3a-generated tasi-RNAs have been shown to be a mobile signal involved in SAR by repressing the expression of auxin response factors and enhancing plant antiviral responses, though further investigation is warranted [208]. In addition, an important area remains, namely the study of the impact of climate change on the spread of viruses [30] and the prediction of the emergence of new strains.

One of the most promising areas in the research and fight against plant viruses, including potato viruses, is the use of CRISPR/Cas-based genome editing technologies. These tools allow you to precisely “switch off” or reconfigure genes involved in virus reproduction, its spread, or plant sensitivity without disrupting normal crop growth. For example, in a study by Zhang et al., the CRISPR/Cas13a system was successfully used to obtain potato lines resistant to the PVY virus. Scientists introduced the Cas13a protein and guide RNAs (sgRNA) into plants, “targeted” to conservative regions of the virus genome (P3, CI, NIb, and CP). After *Agrobacterium*-mediated transformation, resistance was tested using real-time PCR, ELISA, and immunolabeling. As a result, it was possible to significantly reduce the viral load, and the level of protection directly depended on the expression of Cas13a and sgRNA. Moreover, the system worked against several PVY strains at once: PVY^0^, PVY^N^, and PVY^O^ [209].

In another study, Noureen et al. (2022) [210] used CRISPR/Cas9 to “switch off” the eIF4E gene, a host protein required for the virus to reproduce. As a result, potato lines with a sharply reduced infection level were obtained. The editing efficiency was confirmed by sequencing, ELISA, and PCR methods.

In addition to Cas9 and Cas13a, systems based on Cas12a (Cpf1) are being actively studied. This enzyme is highly accurate and can cut DNA to form “sticky” ends, which is convenient when editing AT-rich regions, such as viral promoters [151]. Uranga et al. (2021) [211,212] implemented an original system for delivering Cas12a and sgRNA using PVX and TEV viruses—without incorporating foreign DNA into the plant genome. Although the work was not carried out on potatoes, its results open up possibilities for using this approach in potato growing.

Another direction is point editing: base editing and prime editing methods allow for the change of individual nucleotides without breaking DNA. This is especially important when working with sensitive genes, such as eIF4E, where it is important to change the function but not disrupt the protein as a whole [213].

For fine regulation of expression, CRISPRi (inhibition) and CRISPRa (activation) systems are used based on “inactive” forms of Cas proteins (for example, dCas9 or dCas13), which do not cut DNA but affect gene activity. In addition, epigenetic editors such as dCas9-TET and dCas9-DNMT that alter the methylation level in promoters are being developed, “switching off” unwanted genes without mutations in their code [214,215].

A very promising approach is to combine CRISPR and RNA interference (RNAi) within the framework of the so-called stacked resistance concept. Such plants both produce vsiRNA against the virus and express Cas proteins, increasing the effectiveness of protection against rapidly mutating viruses. Similar approaches have already proven themselves in potatoes and other crops [216].

Some scientists are also working on strategies to prevent the transmission of the virus through insects. For example, the PVY virus has a protein called HC-Pro that interacts with receptors in the mouth of the aphid *Myzus persicae*, ensuring its infection. In response, it is proposed to create plants that produce “baits”—proteins that block HC-Pro and interfere with the transmission of the virus. It is also proposed to use CRISPR or RNAi to “turn off” the genes of viral receptors in the salivary glands of aphids, reducing their ability to spread viruses [109].

Finally, another direction is to change plant signals that affect insect behavior. Viruses often change the composition of volatile organic compounds (VOCs), making the plant more attractive to vectors. Changing the functioning of VOC biosynthesis genes using CRISPR can reduce the attractiveness of the plant to aphids and slow the spread of infection in the field [109].

## Figures and Tables

**Table 1:** Characteristics of potato virus Y, the potato leafroll virus, and potato virus X.

Characteristic	PVY (Potato Virus Y)	PLRV (Potato Leafroll Virus)	PVX (Potato Virus X)
Family	*Potyviridae* [43,44]	*Solemoviridae* [45,46,47,48,49]	*Alphaflexiviridae* [50,51,52]
The shape of viral particle	filamentous [53]	icosahedral [46,48]	filamentous [50,51,52]
Genome	+ssRNA (single-stranded and positive-sense) [53]	+ssRNA (single-stranded and positive-sense) [49]	+ssRNA (single-stranded and positive-sense) [50]
Genome size	9.7 kb [53,54]	5.3–5.7 kb [45,46,55]	6.4 kb [50]
Genome end’s structure	VPg at the 5′ end and poly(A) tail [56]	VPg at 5′ and structured 3′-UTR (no polyA) [57,58]	5′ cap and poly(A) tail [52,55]
Site of virus replication in plant	cytoplasm and associated with vesicular structures and endoplasmic reticulum [59]	cytoplasm of phloem cells and replication strictly limited to phloem cells [45]	cytoplasm and associated with ER membranes [60,61]
Replication mechanism	minus-strand RNA serves as an intermediate template for genomic plus-strand RNA synthesis; synthesis of subgenomic RNA is limited [62]	minus-strand RNA serves as an intermediate template for genomic plus-strand RNA synthesis; subgenomic RNAs are formed for gene expression [45,46,63]	minus-strand RNA serves as an intermediate template for genomic plus-strand RNA synthesis; subgenomic RNAs are formed for gene expression [52]
Local movement in the plant	moves through plasmodesmata using viral movement proteins (MPs) [64,65,66]	a specific RTP domain for movement through the plasmodesmata [46,48]	triple gene block (TGB) proteins facilitate transport through the plasmodesmata [55,60]
Systemic movement in the plant	movement through the phloem; uses HC-Pro to facilitate cell-to-cell and systemic movement [44,67]	strictly phloem-limited and movement via sieve elements (CP and RTPD proteins) [46,68]	primarily through the phloem and occasionally detected in the xylem (TGB and CP proteins) [52]
Modes of viral transmission	vegetative propagation, insect vectors (aphids), and mechanical contact [44,69,70,71]	vegetative propagation and insect vectors (aphids) [58,69,72]	vegetative propagation and mechanical contact [51,61]

**Table 2:** Potato plants response to the viruses (PVY, PLRV, and PVX).

Response Mechanism	Potato Virus Y (PVY)	Potato Leafroll Virus (PLRV)	Potato Virus X (PVX)
RNAi mechanism	vsiRNA production against HC-Pro and CP [146]; key host factors DCL2, DCL4, AGO1, and RDR6; HC-Pro suppresses siRNA accumulation and AGO1 activity [44].	vsiRNA generated against CP, P1, and RTP (limited studies) [147]; host factors likely include DCL2, DCL4, AGO1, and RDR1/6; CP and/or RTP may act as suppressors (mechanism unclear) [147,148].	vsiRNA production targets P25 and CP [51,61,104]; host factors include DCL4, AGO2, and RDR6; P25 is a potent suppressor of local and systemic silencing [149,150,151].
R gene-mediated resistance	HR (*Ny*-1 and *Ny*-2) and ER (*Ry*_sto_, *Ry*_adg_, and *Ry*_chc_) [144,145]; *Ny*-1 decreases efficiency at >24 °C [142,143].	*R* gene-mediated (*Rl*_adg_, *PLRV.1*, *PLRV.2*, *PLRV.3*, and *PLRV.4*); SAR activation [13,152,153,154].	HR (*Nx* and *Nb*) and ER (*Rx*1 and *Rx*2) [51,52]. ER is stable at various temperatures [142].
PR gene expression	strong upregulation of *PR*-1b and other *PR* genes via SA signaling [155]; linked to SAR and MAPK activation [142,156].	no confirmed data for PLRV.	expression of *PR* genes observed, especially under stress or co-infection [151,157,158]; varies by host genotype and viral load [51,149,157,158].
Plant hormones response	SA increases [155] and ABA accumulation observed [156].	SA increases and JA/ET signaling suppressed [72].	slight increase in SA and ABA levels modulated [159]; ISR induced by endophytic *Bacillus* strains [160].
MAPK signaling pathway activation	activated [156]; contributes to HR and PR gene expression (PR-1b) [155].	not well characterized.	activated via P25-induced stress; contributes to ROS production and HR [149,151].
Impact of endophytic bacteria	*Bacillus* strains induce ISR, reducing the viral load [161].	not specifically studied	*Bacillus* strains induce ISR, reducing the viral load [160,161].
